# Case of Scirrhus Pylori and Ulceration of the Stomach

**Published:** 1830-01-01

**Authors:** 


### Scirrhus of the Pylorus and Ulceration of the Stomach.

Mr. Waldron of Bath has related an interesting case of this melancholy disease, in the last number of our Midland contemporary, which deserves notice on account of one or two curious circumstances connected with it.

The patient was a Mr. Anthony, æt. 50, commercial traveller of Bath, addicted to drinking spirits, who applied to Mr. Waldron on the 2d December, 1828, with much debility, loss of appetite, uneasiness at the pit of the stomach, and frequent vomiting. The face was sunk and sallow, the pulse extremely weak, the bowels very irregular. Till within the preceding eight months he had enjoyed an uninterrupted state of good health. No fulness nor tenderness was detected on examining the abdomen, and some purgative medicines were prescribed. These, however, failing to effect any benefit, Mr. Waldron inquired further, and discovered that the sickness generally occurred from an hour and a half to three hours after eating, and that the matters brought up were dark-coloured, grumous, and more than commonly offensive; the evacuations were imperfect and scanty, and for some length of time a copious and sound stool had not been voided. Calomel and hemlock, leeches, and saline aperients were now prescribed, but without any benefit, and on the 8th of January, being informed that some difficulty was experienced in administering the enema, Mr. Waldron examined the rectum himself, and by means of the stomach-pump found that some obstruction did exist, though a quantity of hardened fæces were brought away. Shortly after this a small bougie was introduced with some difficulty, when the patient expressed himself *greatly relieved, and the sickness of the stomach quite subsided.* The bougie was gradually augmented in size, and a pill of three grains of calomel with eight of the pil. rhei comp. prescribed with surprising, though transient good effect. The patient began to sink, became furiously insane, and died on the 10th of February, the sickness never having returned since the use of the bougie.

*Sectio cadaveris*, 48 *hours after death.*—"I examined the body in the presence of Mr. G. Goldstone, surgeon, of this city. Considerable emaciation had taken place in the muscles of the extremities; on cutting through the parietes of the abdomen, an unusual depth of adipose matter was found. In the abdomen, the vessels of the small intestines appeared dark, and congested with blood. I passed a ligature in two points above the cardiac extremity of the stomach, and having divided the part between the ligatures, was proceeding to trace the stomach downwards to its pyloric extremity, when it broke, and extravasated its contents into the abdomen. I next separated the small and large intestines, following them downwards throughout their whole course. Upon laying open the stomach, the cardiac extremity appeared enlarged and thickened, and the pyloric was a complete mass of disease; at that part where a conoidal opening is formed by the termination of the stomach projecting into the duodenum, an enlargement the size of a large duck egg was found; the stomach above this enlargement was ulcerated and thickened, and appeared as a pulpy mass; at the enlargement, the calibre of the part appeared to be nearly obliterated, and below it, the duodenum was ulcerated and thickened for several inches, and exhibited the same appearance of pulpiness, and was so fragile as to break upon the slightest force being used. On cutting through the enlargement at the pyloric extremity of the stomach, the centre exhibited a scirrhous hardness; in one part, there was a dark discolouration, similar to what is seen in scirrhous enlargement of the breast, prior to its passing into the ulcerative stage; on cutting into it, a dark coloured sanies escaped, which was imbedded in a tubercular cyst. The rest of the viscera of the abdomen exhibited no morbid appearance, excepting the colon and rectum, which were in several parts so much contracted, as to reduce the calibre of the intestinal tube to the size of the smallest rectum bougie. In the sigmoid flexure of the colon, these contractions were very apparent, and the fæces were with considerable difficulty made to pass these points, by pressing the finger and thumb above, and propelling the fæces forward.

"When the colon and rectum were laid open, beginning above the left iliac region, the intestine appeared in many parts thickened and contracted; in other respects no morbid appearance was observed. In the head, the vessels of the brain appeared dark and congested with blood; the tunica arachnoidea was very much thickened, and had become dense and obscure; in different parts, especially on one side, deposits of coagulable lymph were observed; a larger quantity of fluid than what is common, was found in the ventricles; in other respects, the brain exhibited no morbid appearances."

As Mr. Waldron justly observes, the marked relief from the passage of the bougie, and cessation of the sickness are remarkable, and prove the intimate though mysterious consent and sympathy between the different portions of the alimentary canal. The absence of pain on pressure, though uncommon, is occasionally met with, and no necessity exists for admitting the hypothesis advanced to account for the circumstance by the author—viz. the quantity of fat in the abdominal parietes. We have seen the same absence of pain on pressure, where no such condition obtained.

